# Patient Source of Referral Is a Key Determinant of Subsequent Retention in Care for Young Chronic Hepatitis B Patients

**DOI:** 10.1111/jvh.14059

**Published:** 2025-01-09

**Authors:** David Mutimer, Maxine Brown, Jacqueline Logan, Chayarani Kelgeri

**Affiliations:** ^1^ Liver and Hepatobiliary Unit Queen Elizabeth Hospital, University Hospitals Birmingham NHS Foundation Trust Birmingham UK; ^2^ Institute of Immunology and Immunotherapy, College of Medical and Dental Sciences University of Birmingham Birmingham UK; ^3^ Liver Unit Birmingham Children's Hospital, Birmingham Women's and Children's NHS Foundation Trust Birmingham UK

**Keywords:** care continuum, hepatitis B, liver, viral hepatitis

## Abstract

Hepatitis B elimination objectives can only be realised if new patient linkage to care is matched by long‐term patient retention in care. We previously showed in adult chronic hepatitis B (CHB) patients that retention in care was inferior in younger patients and in patients from non‐Asian ethnicities. The present study explores further the rates and determinants of loss to follow‐up in a cohort of 271 young patients (aged 16–21 years at baseline). 16% of patients were lost to follow‐up after a single consultation, and retention in care at 5 and 10 years was 53.7% and 45.9%, respectively. Retention in care was strongly associated with the source of patient referral and was superior for patients referred from the antenatal clinic and those transitioned from paediatric care (68% retention at 5 years for both sources) compared with those from “other” sources (36% at 5 years). In multivariate analyses, patient source of referral and distance of current residence from the Hepatitis Outpatient Clinic were the significant determinants of loss to follow‐up. Retention in care may have been promoted by the transition process for those diagnosed in childhood and by the repeated referral from the antenatal clinic of women who had multiple pregnancies during the observation period. Only 20% of asylum seekers and referrals from genitourinary clinics were retained in follow‐up at 10 years from baseline. This identifies a group of patients who do not access medical care, cannot benefit from treatment, and who may constitute a long‐term public health risk.

Chronic hepatitis B (CHB) is a life‐long infection for most patients. In many countries, including the United Kingdom (UK), CHB patients are managed in secondary care. Thus, specialist Hepatology and Infectious Diseases clinics have accumulated large cohorts of patients with CHB.

The continuum of care for patients with HCV infection has received a lot of attention during the past decade [1]. For many HCV patients, the continuum of care can be completed by administration of a short curative course of antiviral treatment. In contrast, a minority of infected patients eliminate HBV infection, either with or without antiviral therapy. Patients on treatment and untreated CHB patients need to be managed and retained in specialist outpatient (OP) care. There is emerging evidence about rates and determinants of linkage to care for HBV‐diagnosed patients [2, 3, 4, 5]. However, to date, little attention has been paid to the important issue of CHB patient retention in the outpatient system [6, 7, 8]. There may be special challenges. In western centres, the majority of CHB patients are first‐generation migrants from countries of higher HBV prevalence [9]. It is likely that adherence with long‐term follow‐up may differ between ethnic groups. Also, a significant proportion of the CHB population is diagnosed at a young age, which is known to be associated with a higher risk of loss to follow‐up of many conditions in the long term. Oorschot and colleagues examined determinants of retention in care of CHB in the Netherlands and found that patient age at presentation (superior retention associated with older age) and patient ethnicity (superior retention for Asian versus Caucasian and African patients) were possible determinants of retention in care.

In a recently published analysis of a large adult multi‐ethnic cohort of CHB patients attending QEH, we showed that retention in care was 73.2%, 64.1%, 55.6% and 50.3% at 5, 10, 15 and 20 years of follow‐up. Also, (South) Asian ethnicity and older age at baseline were associated with lower rates of loss to follow‐up [7].

The aim of the current study was to explore in greater detail the outcomes of young patients (21 years of age or younger at baseline visit) and to explore the possibility that the source of patient referral to the adult clinic might be a determinant of retention in care. We wondered if the antecedent period, often prolonged, of management in the paediatric setting (and with a transition process to adult care) might be associated with a reduced likelihood of loss to follow‐up in comparison with those patients who are diagnosed for the first time in adulthood. We hoped to identify patient demographic characteristics that might distinguish higher from lower‐risk patients. This, in turn, might enable the targeted implementation of measures to reduce the rate of patient loss and to maintain patients in clinic follow‐up for the lifetime of the infection.

We also examined the rates of HBeAg and HBsAg loss during prolonged follow‐up of the same cohort.

## 1 Patients and Methods

The Queen Elizabeth Hospital (QEH) provides care to the local Birmingham population and provides specialist liver services to the West Midlands region of England. Most CHB patients are referred from local sources, including primary care, genitourinary medicine (GUM) clinics, and antenatal clinics (ANC). All pregnant women are tested for HBsAg in all pregnancies at the 2 large Birmingham maternity hospitals. Each antenatal clinic employs a specialist viral hepatitis liaison midwife whose role is to link with the QEH viral hepatitis service. This role has been established for more than 20 years. In addition, the care of children with CHB infection is transferred from Birmingham Children's Hospital (BCH) to the QEH Liver Clinic at about 18 years of age. The transition process for young children has been consistent during the 22‐year period of observation described in the present analysis. At the age of transition, young patients are reviewed by the adult hepatologist with the paediatric team at the BCH. Thereafter, patient review is entirely devolved to the adult hospital (QEH) and its multidisciplinary team. Acknowledging the specific challenge that patient relocation for tertiary studies and employment poses to retention in care, a flexible approach to the scheduling and rescheduling of clinic appointments (e.g., to coincide with university vacation) is adopted. In addition, patients are asked to inform us if and when permanent geographical relocation might occur. In that case, a summary of care is provided to the new general practitioner and referral to local specialist services is recommended.

These sources of referral have been established for many years, and the adult HBV population attending the QEH is large and reflects the ethnic diversity of the local population. The 2021 UK National Census found that 48.7% of the people of Birmingham were white, 29.9% were Asian (referring to the South Asian population), 1.1% were Chinese, and 10.9% were Black (see census.gov.uk, acquired 16 January 2023).

At the time of hospital registration, patients are asked to identify their ethnicity, though some patients (about half) decline to do so. For NHS administrative purposes and used in our analysis, patient ethnicity was categorised as Asian (includes Bangladeshi, Pakistani, and Indian), Black (includes African or Caribbean), Chinese (including other East Asians), and White. Patients of mixed ethnicity were not included in analyses that examined the association of ethnicity with retention in care and serologic outcomes.

CHB patients were managed in dedicated adult outpatient clinics according to agreed protocols. For patients with HBeAg‐positive infection and hepatitis, review with blood tests was undertaken every 6 months. For patients with HBeAg‐negative serology, review was 6‐monthly for those with chronic hepatitis and annually for those with chronic infection. At each outpatient attendance, serum HBsAg, HBeAg, anti‐HBe, and HBV DNA were measured. If a patient failed to attend a scheduled outpatient appointment, then another appointment (typically within 3 months of the first failed appointment) was offered to the patient. Failure to attend on a second occasion led to patient discharge to the general practitioner (with an offer to resume specialist care if and when the patient might wish to do so). Thus, the absence for a period of 18 months or longer should reliably identify a patient who has missed at least 2 consecutive outpatient appointments and is no longer in follow‐up.

Patients were classified as “lost to follow‐up” under the following circumstances:

- No visit in the 18 months prior to the cut‐off date of 9 November 2022.
- No visit in the 18 months prior to the death date.

Clinic attendance data were censored (on the date of the most recent outpatient visit) in the following circumstances:

- Most recent visit within the 18 months prior to 9 November 2022.
- Most recent visit within 18 months prior to registered date of death.
- HBsAg negative result at most recent visit (in these cases, the patient was assumed to be discharged instead of lost to follow‐up).

Eligible patients were those with a positive HBsAg blood test performed by the QEH laboratory during the period 1 January 2001–9 November 2022 and aged 21 years or younger at the time of the baseline QEH visit. Relevant data were extracted from multiple electronic sources and included basic demographic information, laboratory data, and clinical disease and event healthcare coding. In addition, individual patient electronic and paper files were interrogated to identify the source of patient referral and the patient ethnicity (if not already specified by self‐declaration in the electronic patient record). All data were collected and up to date on 9 November 2022. Statistical analyses were performed with the Excel add‐in Statpages, and *p*‐values smaller than 0.001 are simply stated as *p* < 0.001. The study was approved by the QE Hospital's Research and Development Department (code CARMS‐18094).

## 2 Results

We identified 3770 individual patients with at least one positive HBsAg test between 1 January 2001 and 9 November 2022. Two hundred and seventy one of these were aged 21 years or younger at the time of the baseline HBsAg test. These are shown in Table 1, which describes the cohort according to ethnicity and baseline HBeAg/anti‐HBe status. The cohort of 271 included 127 males and 144 females, with a mean age of 19 years, a median age of 19 years, and range of 16–21 years at baseline.

**TABLE 1:** HBeAg and anti‐HBe status at baseline according to ethnic group and the percentage in each group who were HBeAg‐positive at baseline. A high proportion of patients (103/271) had persisting HBeAg‐positivity at baseline.

Ethnicity	Number	HBeAg/anti‐HBe					HBeAg‐pos/total (%)
		+/+	+/−	−/+	−/−	Incomplete or not known	
Asian	126	2	48	74	2	0	39.7%
Black	68	0	13	52	1	2	19.7%
Chinese	39	4	23	11	1	0	69.2%
White	34	1	10	22	0	1	33.3%
Mixed	4	1	1	1	0	1	66.6%
Total	271	8	95	160	4	4	38.6%

The HBeAg/anti‐HBe status was known for 267 patients at baseline. Overall, HBeAg status at baseline was positive for 103/267 (38.6%). Black patients were least likely (19.7%) and Chinese patients were most likely (69.2%) to be serum HBeAg‐positive at baseline.

### 2.1 HBeAg and HBsAg Loss During Follow‐Up

One hundred and three patients were HBeAg‐positive at baseline, and 25 patients lost HBeAg during follow‐up. The 5‐ and 10‐year rates of loss were 28% and 42%, respectively (see Figure S1). The rate of HBeAg loss during the first 10 years of observation was approximately linear and occurred at 4%–5% per annum. The subsequent rate appeared to diminish significantly. The rate of HBeAg loss was lower in Chinese versus Black or Asian patients (not achieving statistical significance, though patient numbers are small). The profile HBeAg/anti‐HBe +/+ (observed in 8 patients at baseline) was associated with an increased risk of HBeAg loss. A lower serum HBV DNA measurement at baseline was also associated with increased risk of HBeAg loss (Table S1).

In Kaplan–Meier analysis, there was no difference between male and female sex with respect to rate of HBeAg loss (Figure S1b, log rank *p* = 0.686). We could not demonstrate a statistically significant difference between rates of HBeAg loss versus ethnicity, but the number of events per ethnic group was few (Figure S1c, log rank *p* = 0.932). None of the pairwise comparisons of ethnicity versus rate of HBeAg loss were statistically significant (not shown).

Eight patients became HBsAg‐negative during follow‐up. HBsAg loss at 10 years was 6%, predictably low for a population that was young and included a large proportion of patients who were HBeAg‐positive at baseline (Figure S2).

### 2.2 Patient Retention in Care

**FIGURE 1:**
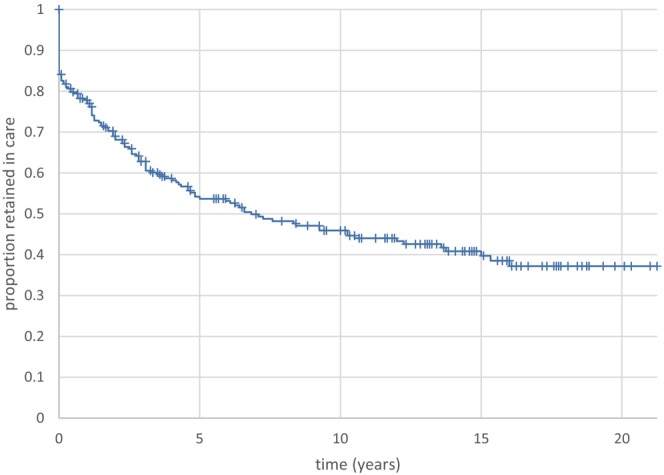
Patient retention in follow‐up after baseline attendance. The initial vertical drop (16%) reflects the loss to follow‐up of patients after a single outpatient attendance. Thereafter, loss to follow‐up occurs at a greater rate during the first 5 years in comparison with the second 5‐year period of follow‐up.

**FIGURE 2:**
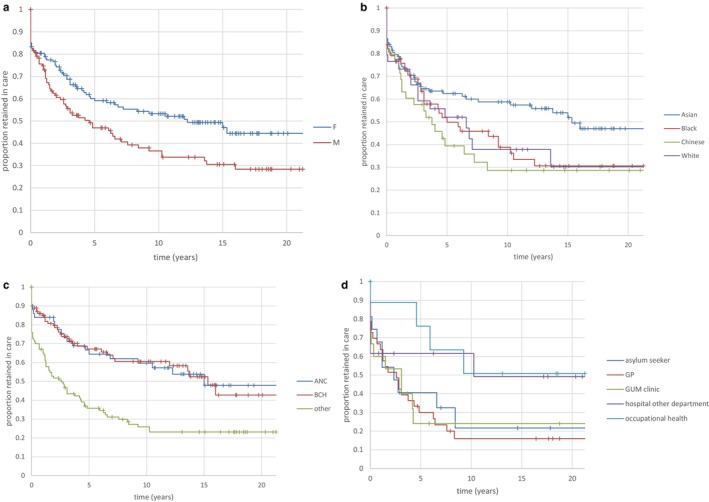
(a) Kaplan–Meier analysis of risk for loss to follow‐up versus patient sex. Males are much more likely to be lost, with fewer than 50% of males retained in care 5 years after baseline attendance (log rank *p* = 0.015). F = female, M = male. (b) Kaplan–Meier analysis of risk for loss to follow‐up versus patient ethnicity. Overall, the 4‐way comparison did not achieve statistical significance (log‐rank *p* = 0.079). However, pairwise comparisons (Table S2) showed that retention in care of Asian patients was statistically superior to retention of Chinese patients (HR = 1.7432). (c) Kaplan–Meier analysis of risk for loss to follow‐up versus patient referral source (log rank *p* < 0.001). Retention in care was clearly superior for patients referred from BCH (adult transition) and the local antenatal clinic. However, the “other” group included quite heterogeneous referral sources, some sources displaying better retention than others (see Figure 2d). ANC = antenatal clinic, BCH = Birmingham Children's Hospital. (d) Kaplan–Meier comparison of retention in care versus source of referral for 85 patients referred from sources other than BCH and ANC (log rank *p* = 0.166). GP = general practitioner, GUM = genitourinary medicine. For 18 patients, referral source could not be verified. Asylum seekers (*n* = 16), GP referrals (*n* = 33), GUM clinics (*n* = 15), in‐hospital referrals from non‐liver departments (*n* = 12), occupational health departments (*n* = 9).

For this analysis, all 271 patients were included. Those patients with the most recent outpatient attendance within 18 months of 9 November 2022 were classified as retained in follow‐up, and the duration of follow‐up was the interval from baseline to the most recent outpatient attendance. Those patients who died during follow‐up (*n* = 2), and whose last OP attendance was within 18 months of death, were also considered retained in follow‐up, and the duration of follow‐up was censored at last OP attendance. Patients who cleared HBsAg (*n* = 8) were treated as discharged, not lost to follow‐up, and their duration of follow‐up was from baseline to last OP attendance. According to this algorithm, 134/271 (49.4%) were retained in care, and 137/271 (50.6%) were lost to follow‐up. The survival curve representing the proportion retained in follow‐up is shown in Figure 1. 16% of patients attended the outpatient clinic on a single occasion only, 53.7% were retained at 5 years, and 45.9% at 10 years. Thus, the risk of loss to follow‐up is maximal during the first 5 years after baseline, and adherence with follow‐up appears better beyond 5 years (only 8% attrition during the second 5‐year period of follow‐up). Baseline characteristics of those who were retained in care are compared with characteristics of those who were lost to follow‐up in Table 2. Retained patients were more likely to be female, more likely to have South Asian ethnicity, and had a greater likelihood of being referred from BCH or from the local ANC than from other sources. The association of patient sex, ethnicity, and patient referral source with retention in care was explored further by Kaplan–Meier analyses (Figure 2a–c). Patient sex (Figure 2a, log rank *p* = 0.015) and source of referral (Figure 2c, log rank *p* < 0.001) achieved statistical significance. Patient ethnicity (Figure 2b) nearly achieved statistical significance, with Asian patients having the best retention in care (log rank *p* = 0.079). Pairwise comparison of ethnicities (Table S2) showed that Asian patients had a statistically greater chance of being retained in care in comparison with Chinese patients (HR 1.7342). The patient drop‐out rate following a single QEH clinic attendance was 11% for those referred from BCH and ANC but 23% for those referred from other sources. At 5 years from the baseline visit, 68% of BCH and ANC referrals maintained follow‐up, but only 36% of those from other referral sources did. The association of referral source with patient retention in care was highly significant (Figure 2c, log rank *p* < 0.001), with reduced retention in care observed for the group of patients referred from sources other than BCH and ANC. The other referral sources are compared by Kaplan–Meier analysis in Figure 2d, though the number of patients in some subgroups is quite small. However, it appears that patients referred to the clinic from occupational health (OH) sources (*n* = 9) had an increased chance of retention in care compared with referrals from General Practitioners (*n* = 33) and GUM clinics (*n* = 15) (see Table S3 for the pairwise comparisons; hazard ratio (HR) = 2.7395 for OH vs. GP and 2.6398 for OH vs. GUM).

**TABLE 2:** Determinants of retention in care for the cohort. Retention was associated with female sex, with patient ethnicity (most likely in Asian ethnicity patients), and with referral from BCH or ANC (versus other sources).

Number	Retained	Lost to f‐up	Statistics
	134	137	
Sex (male: female)	55:79	72:65	*p* = 0.058^a^
Baseline age (years) (mean, median, range)	19.5, 20, 16–21	19.4, 20, 16–21	*p* = ns^b^
Ethnicity			
Asian	74 (58.7%)	52	*p* = 0.053^a^
Black	30 (44.1%)	38	
Chinese	14 (35.9%)	25	
White	15 (44.1%)	19	
Mixed	1 (25%)	3	
Referral source			
BCH	76 (65.0%)	41	*p* < 0.001^a^
Antenatal (ANC)	28 (56%)	22	
Other	30 (28.8%)	74	

^a^ Chi‐squared test.

^b^ Mann–Whitney *U* test.

The imbalance of patient sex distribution versus source of referral and the interaction of these parameters needs to be considered. Univariate analysis (Table 2) shows that patient sex may be a determinant of retention in care, with female gender determining superior retention. Understandably, all ANC referrals were female. For BCH referrals, 60/117 (51.3%) were female, but only 34/104 (32.7%) of referrals from other sources were female. To explore this and other potential interactions, Cox multivariate analysis and binary logistic regression analysis (the latter also including the distance of current patient residence from the QE OP clinic) were undertaken, and the results are shown in Tables 3 and 4. The analyses included 267 patients, excluding 4 patients with mixed ethnicity.

**TABLE 3:** Cox multivariate analysis of determinants of retention in care, including the baseline characteristics (sex and ethnicity) and source of patient referral. Asian ethnicity is associated with superior retention in care compared with Chinese and White patients (*p* = 0.098 and *p* = 0.057, respectively). Patients referred from sources other than BCH and ANC have poor retention in care (*p* < 0.001).

Parameter	Number	Risk ratio (RR)	LCL	UCL	*p*
Sex					
Female	142	ref			
Male	125	1.070	0.715	1.601	0.743
Ethnicity					
Asian	126	ref			
Black	68	1.262	0.822	1.937	0.287
Chinese	39	1.503	0.927	2.435	0.098
White	34	1.678	0.985	2.858	0.057
Referral source					
BCH	115	ref			
Antenatal	50	1.027	0.590	1.786	0.925
Other	102	2.207	1.453	3.352	< 0.001

**TABLE 4:** Binary logistic regression analysis, including baseline characteristics (sex and ethnicity and source of referral to liver clinic) and distance from patient home address to liver clinic (only the most recent address was available from electronic record, i.e., baseline address was not available for this analysis). In this analysis, “other” referral sources and longer distances of current residence from the clinic were associated with inferior retention in care.

Parameter	Number	Odds ratio (OR)	LCL	UCL	*p*
Sex					
Female	142	Reference			
Male	125	1.266	0.690	2.322	0.446
Ethnicity					
Asian	126	Reference			
Black	68	1.129	0.578	2.205	0.723
Chinese	39	1.769	0.789	3.968	0.166
White	34	1.399	0.594	3.292	0.442
Referral source					
BCH	115	Reference			
Antenatal	50	1.760	0.810	3.822	0.153
Other	102	3.960	2.149	7.300	< 0.001
Distance from clinic	267	1.008	1.002	1.015	0.010

In the time‐dependent Cox multivariate analysis (Table 3), patient referral source was the only statistically significant determinant of loss to follow‐up, with a risk ratio (for “other sources” vs. BCH referrals) of 2.207. In the same analysis, retention in care did not differ between patients transitioned from BCH versus those referred from ANC. Chinese and White ethnicities might also determine greater risk of loss to follow‐up (in comparison with South Asian patients), with *p*‐values of 0.098 and 0.057, respectively. Patient sex as a determinant of retention in care was not significant in this multivariate analysis.

Table 4 extends the analysis by examining the association of retention in care with the patients' current (at 9 November 2022) home address (i.e., calculating the distance from home to clinic). Because this parameter is not baseline (and will be different from the patient address at baseline in many cases), it cannot be included in the Cox analysis, so bivariate regression analysis was undertaken instead. Once again, there was a strong association of patient referral source with loss to follow‐up. In addition, loss to follow‐up was associated with distance of residence from clinic (odds ratio (OR) 1.008, so nearly 1% increase in risk for every additional kilometre distance from clinic). Patient sex was not statistically significant in this analysis.

### 2.3 Patient Characteristics at Time of Loss to Follow‐Up

One hundred and thirty seven patients were lost to QEH follow‐up. The average age at the last outpatient visit for these patients was 22 years, the median 21 years, and the range 16–36 years. At the time of loss to follow‐up, 32 patients maintained HBeAg‐positivity and 105 were anti‐HBe positive. One hundred and twenty one patients had serum HBV DNA measured at the last visit: 67 had HBV DNA < 1000 IU/mL, 20 had a titer between 2000 and 10,000 IU/mL, 14 between 10,000 and 100,0000 IU/mL and 20 patients had a titer > 1,000,000 IU/mL. Fifteen patients were taking suppressive antiviral treatment at the time of the last outpatient review (tenofovir 9 patients, entecavir 6 patients).

## 3 Discussion

WHO elimination objectives for HBV include an increase in case ascertainment and an upscaling of antiviral therapy for those in care [10, 11]. Case ascertainment needs to be matched by patient retention in care to maintain long‐term supervision of this chronic disease.

In a generic study of the problem of outpatient clinic non‐attendance in the UK, Philpott‐Morgan and colleagues measured rates of unkept outpatient clinic appointments in hospitals in England [12]. Across 50 busy hospitals, the rate of unkept appointments was 8% (range 3.9%–14.8%), and they found that the speciality with the highest non‐attendance rate was hepatology (17%). Predictors of non‐attendance included past non‐attendance and younger age.

Studies that examine retention in care for CHB patients are few, include only small numbers of patients, and lack long‐term follow‐up. We have used electronic data capture from clinical and healthcare administrative resources to examine the rate and determinants of CHB patient loss to follow‐up. Electronic datasets with longitudinal information are ideal for analysis of time‐dependent outcomes [7, 13]. The 18‐month cut‐off that we used to define loss to follow‐up is suited to the follow‐up schedule implemented in our adult QEH clinic. Others have applied different definitions and cut‐offs [14, 15, 16].

Published estimates for retention in care of CHB patients are few. Magnasco and colleagues looked at a cohort of 143 CHB patients and found retention in care for 87%, 71% and 60% at 1, 3 and 5 years, respectively [14]. They found that African origin and the presence of HIV coinfection were positively associated with retention in care. Young and colleagues looked at linkage and retention in care for an immigrant refugee population attending 3 clinics in the USA [17]. The crude rates of retention in the 3 clinics were only 11%, 12% and 21% respectively. Lieveld and colleagues found that 161 (47%) of 343 consecutive CHB patients were lost to follow‐up at some stage, that immigrant refugees had the greatest risk, and that older patients and those who received antiviral treatment had a lower risk [15]. Beekmans and colleagues found that 356 (64.5%) of 552 CHB patients lacked outpatient appointments for follow‐up [16]. Tang and colleagues evaluated linkage to care and retention in care of a multi‐ethnic cohort of adult CHB patients. In a cohort comprising 72.7% Asian (East Asian) patients, linkage to CHB care was achieved for 63.2% of patients, and 69.6% of those patients were retained in care (defined as completion of 2 additional encounters with an HBV provider). In this study, patient retention was better for men than for women and was superior for (East) Asians versus non‐Asian patients [18].

These reports are consistent with our observations that CHB infection is associated with poor rates of retention in care in the long term, that patient ethnicity may affect the chance of retention in care, and that the source of patient referral might also be relevant.

Our analyses identify a difference between the rates of retention in care between South Asian patients and those of other ethnicities. This difference may be partly explained by rates and patterns of population internal migration in the UK [19]. South Asian patients are least likely to undergo internal migration. Our analysis does not tell us if a patient is lost to all CHB follow‐up or simply lost to QEH CHB follow‐up. Further studies will be required to establish this important difference.

Our previously published analysis of the rate and determinants of loss to follow‐up showed that young age at baseline, male sex, and non‐Asian ethnicity were determinants of loss to follow‐up [7]. The current analysis extends those observations to focus specifically on CHB patients who were referred and seen for the first time in clinic as young adults (21 years or younger at baseline).

We wondered if management of CHB in childhood with a planned transition to adult Hepatology services might be associated with superior long‐term adherence in comparison to those patients who were diagnosed with CHB for the first time in early adulthood. It has been proposed that non‐adherence of transition patients may be promoted when chronic disease is asymptomatic [20]. Another promoter of poor adherence in transition patients may be that adult services rarely provide families with the level of support that was available during paediatric care [21]. Guidance and expert reviews on the design and implementation of the transition process have been published [20, 21, 22, 23, 24]. However, objective evidence that future adherence is enhanced by the transition process is lacking. Indeed, a Cochrane analysis designed to examine published evidence of benefit for formal transition failed to show that transitional care programs made a difference in health status, quality of life, or patient well‐being [25]. The impact of transition on long‐term adherence to CHB follow‐up has received almost no attention. Recently, Fisayo and colleagues followed 21 CHB patients who transitioned from paediatric to adult care at a median age of 18 years. In this analysis, there was no loss to follow‐up during a median period of 4.6 years [26].

Our analysis of patient referral source versus retention in care may provide an indirect measure of the benefit of the transition process from BCH to QEH. The study population included 117 patients who underwent transition to QEH after management in childhood at BCH. Despite the transitional arrangements, 11% of BCH patients attended only a single follow‐up appointment in the adult hospital. However, patient retention in care at 5 years after the first visit was 68%. For comparison, the second largest referral source was the local ANC (50 young women). Retention in care for this cohort (whose new diagnosis was made at the first antenatal visit) was identical to the BCH transition patients. Examining the risk for loss to follow‐up from baseline, however, does not take account of factors that may actively promote retention in care during longer‐term follow‐up. For instance, repeated pregnancies can maintain CHB women in long‐term follow‐up. At least 10 of the ANC cohort had more than 1 pregnancy during the follow‐up period of the study. Of those 10 women, 10 had a second, 5 had a third, and one patient had a fourth pregnancy during follow‐up. Each of the 16 additional pregnancies created an opportunity for re‐referral to the CHB clinic for those that may have been lost to follow‐up. Indeed, re‐engagement after a gap in CHB follow‐up (greater than 18 months absence) was seen on 5 occasions in 4 patients, and 2 of the 4 remain in long‐term follow‐up. Despite this pattern of loss and re‐engagement, retention in care of ANC patients was not superior to retention of transition patients.

The “other” source of referral is a heterogeneous group, but dominated by male patients, frequently asylum seekers, who are screened in general practice or after self‐referral to local GUM clinics. Most were lost to local follow‐up, and it seems unlikely that maintenance of CHB care is perceived as a priority by the patient. It may be difficult to improve long‐term adherence with follow‐up of these young, predominantly male patients. However, greater effort is required to address the attrition of the transition and antenatal patients. In particular, 16% of these patients were lost to follow‐up after a single visit to QEH adult hepatitis services. A more comprehensive and engaging first visit, with more time for health education, might be helpful. For those patients who relocate, often for tertiary education and other career choices, a more flexible approach to follow‐up may be required. For all patients, some sort of look‐back engagement process, as is promoted widely for people with untreated hepatitis C infection, should be considered.

At the time of loss to follow‐up, 32/137 patients were still HBeAg‐positive, and 15 had an indication for antiviral treatment. Thus, a significant proportion of lost patients have high levels of HBV replication and have a risk for the development of significant liver disease, including liver cancer, in the long term. In addition, these patients pose a significant public health risk, particularly with risk for sexual transmission of infection. Beekmans and colleagues tracked down and recalled for re‐evaluation a population of 50 CHB patients who had been lost to follow‐up [16]. In this study, 14/50 had an indication for HCC screening. The characteristics of our population at the time of loss to follow‐up and the “lookback” experience of Beekman and colleagues support a strategy of patient identification and retrieval. Such a policy would enable a distinction between those that has been lost to all follow‐up versus a population who have shifted their location of follow‐up. Lookback exercises for the HCV population have become commonplace and should also be undertaken for lost CHB patients.

## Author Contributions

D.M. was responsible for study design, collection of data, statistical analysis, and draughting of the manuscript. M.B. was responsible for data collection and critical review of the manuscript. J.L. was responsible for data collection and critical review of the manuscript. C.K. was responsible for study design and critical review of the manuscript.

## Conflicts of Interest

The authors declare no conflicts of interest.

## Supporting information


Figure S1.

Table S1.

## Data Availability

The data that support the findings of this study are available from the corresponding author upon reasonable request.
