# Supplementary material for: Patient Source of Referral Is a Key Determinant of Subsequent Retention in Care for Young Chronic Hepatitis B Patients
Source: J Viral Hepat. 2025 Jan 9;32(2):e14059. doi: 10.1111/jvh.14059 (PMC11715132; doi:10.1111/jvh.14059)
Supplement: Supplementary file 1 — Figure S1. [file JVH-32-0-s002.docx]

**Figure 2b.** Kaplan-Meier analysis of risk for loss to follow-up versus patient ethnicity. Overall, the 4-way comparison did not achieve statistical significance (log rank p=0.079) However, pairwise comparisons (Supplementary Table 2) showed that retention in care of Asian patients was statistically superior to retention of Chinese patients (HR=1.7432).

**Figure 2c**. Kaplan-Meier analysis of risk for loss to follow-up versus patient referral source (log rank p<0.001). Retention in care was clearly superior for patients referred from BCH (adult transition) and the local antenatal clinic. However, the “other” group included quite heterogeneous referral sources, some sources displaying better retention than others (see Figure 2d). ANC=antenatal clinic, BCH=Birmingham Children’s hospital.

**Figure 2d**. Kaplan-Meier comparison of retention in care versus source of referral for 85 patients referred from sources other than BCH and ANC (log rank p=0.166). GP=general practitioner, GUM=genitourinary medicine. For 18 patients, referral source could not be verified. Asylum seekers (n=16), GP referrals (n=33), GUM clinic (n=15), in-hospital referrals from non-liver department (n=12), occupational health departments (n=9).

**Supplementary Figure 1a**. HBeAg loss for 103 patients who were HBeAg-positive at baseline. HBeAg loss rate for the first 10 years after baseline was approximately 4.5% per annum.

**Supplementary Figure 1b**. HBeAg loss versus patient sex (log rank p=ns). F=female, M=male.

**Supplementary Figure 1c**. HBeAg loss rate versus patient ethnicity (log rank p=ns).

**Supplementary Figure 2**. HBsAg loss rate during follow-up. As predicted for a young population including a significant proportion with HBeAg-positivity at baseline, HBsAg loss was an infrequent event (approximately 0.5% per annum) (note scale on vertical axis starts at 0.7).
