# Supplementary material for: Patient Source of Referral Is a Key Determinant of Subsequent Retention in Care for Young Chronic Hepatitis B Patients
Source: J Viral Hepat. 2025 Jan 9;32(2):e14059. doi: 10.1111/jvh.14059 (PMC11715132; doi:10.1111/jvh.14059)
Supplement: Supplementary file 2 — Table S1. [file JVH-32-0-s001.docx]

|  | | HBeAg loss  n=25 | HBeAg persisting  n=78 | statistic |
| --- | --- | --- | --- | --- |
| sex (male:female) | | 12:13 | 41:37 | p=ns* |
| baseline age (years)  (mean, median,range) | | 19, 19 (16-20) | 19, 18 (16-21) | p=ns@ |
| ethnicity (n=100, unknown for 3) | Asian | 13 (26%) | 37 (74%) | p=ns* |
|  | Black | 4 (30.8%) | 9 (69.2%) |  |
|  | Chinese | 5 (18.5%) | 22 (81.5%) |  |
|  | White | 0 (0%) | 10 (100%) |  |
| anti-e +ve at baseline | | 5/25 | 3/78 | p=0.009* |
| HBV DNA (IU/ml) mean, median (range) | | 115 million, 17 million (54-917 million) | 225 million, 110 million (364->1000 million) | p=0.003@ |
| ALT (U/ml) mean, median (range) | | 67, 37 (12-281) | 66, 36 (11-478) | p=ns@ |

**Supplementary Table 1**. Analysis of the determinants of HBeAg loss in 103 patients who had HBeAg-positivity at baseline. 25 of these became HBeAg-negative during follow-up. Determinants of HBeAg loss versus persistence included concurrent anti-HBe positivity at baseline and baseline serum HBV DNA measurement. *Chi square test, @Mann-Whitney U test.

| **Hazard ratios (retention in care)** | | | | |
| --- | --- | --- | --- | --- |
|  | *HR* | *Std Err* | *LCL (95%)* | *UCL (95%)* |
| "Asian" / "Black" | 0.6939 | 0.2150 | 0.4553 | 1.0577 |
| **"Asian" / "Chinese"** | **0.5766** | **0.2651** | **0.3430** | **0.9694** |
| "Asian" / "White" | 0.6731 | 0.2980 | 0.3753 | 1.2071 |
| "Black" / "Asian" | 1.4411 | 0.2150 | 0.9455 | 2.1964 |
| "Black" / "Chinese" | 0.8310 | 0.2925 | 0.4684 | 1.4741 |
| "Black" / "White" | 0.9700 | 0.3226 | 0.5154 | 1.8254 |
| **"Chinese" / "Asian"** | **1.7342** | **0.2651** | **1.0315** | **2.9156** |
| "Chinese" / "Black" | 1.2034 | 0.2925 | 0.6784 | 2.1348 |
| "Chinese" / "White" | 1.1673 | 0.3579 | 0.5788 | 2.3541 |
| "White" / "Asian" | 1.4857 | 0.2980 | 0.8285 | 2.6643 |
| "White" / "Black" | 1.0310 | 0.3226 | 0.5478 | 1.9402 |
| "White" / "Chinese" | 0.8567 | 0.3579 | 0.4248 | 1.7277 |

**Supplementary Table 2**. Individual Hazard ratios examining risk for loss to follow-up. Chinese patients were more likely to be lost to follow-up than was observed for (South) Asian patients (HR 1.7342).

| **Hazard ratios (retention in care)** | | | | |
| --- | --- | --- | --- | --- |
|  | *HR* | *Std Err* | *LCL (95%)* | *UCL (95%)* |
| "asylum seeker" / "GP" | 0.8699 | 0.3817 | 0.4116 | 1.8381 |
| "asylum seeker" / "GUM clinic" | 0.9027 | 0.4573 | 0.3683 | 2.2123 |
| "asylum seeker" / "hospital other department" | 1.7428 | 0.4513 | 0.7197 | 4.2204 |
| "asylum seeker" / "occupational health" | 2.3829 | 0.4623 | 0.9629 | 5.8974 |
| "GP" / "asylum seeker" | 1.1496 | 0.3817 | 0.5440 | 2.4293 |
| "GP" / "GUM clinic" | 1.0378 | 0.3955 | 0.4780 | 2.2529 |
| "GP" / "hospital other department" | 2.0035 | 0.3884 | 0.9358 | 4.2896 |
| **"GP" / "occupational health"** | **2.7395** | **0.4012** | **1.2477** | **6.0146** |
| "GUM clinic" / "asylum seeker" | 1.1078 | 0.4573 | 0.4520 | 2.7149 |
| "GUM clinic" / "GP" | 0.9636 | 0.3955 | 0.4439 | 2.0919 |
| "GUM clinic" / "hospital other department" | 1.9306 | 0.4630 | 0.7792 | 4.7837 |
| **"GUM clinic" / "occupational health"** | **2.6398** | **0.4738** | **1.0430** | **6.6809** |
| "hospital other department" / "asylum seeker" | 0.5738 | 0.4513 | 0.2369 | 1.3895 |
| "hospital other department" / "GP" | 0.4991 | 0.3884 | 0.2331 | 1.0686 |
| "hospital other department" / "GUM clinic" | 0.5180 | 0.4630 | 0.2090 | 1.2834 |
| "hospital other department" / "occupational health" | 1.3673 | 0.4679 | 0.5465 | 3.4208 |
| "occupational health" / "asylum seeker" | 0.4197 | 0.4623 | 0.1696 | 1.0386 |
| **"occupational health" / "GP"** | **0.3650** | **0.4012** | **0.1663** | **0.8015** |
| **"occupational health" / "GUM clinic"** | **0.3788** | **0.4738** | **0.1497** | **0.9587** |
| "occupational health" / "hospital other department" | 0.7314 | 0.4679 | 0.2923 | 1.8298 |

**Supplementary Table 3**. This analysis included 103 patients, classified in the discussion as “other sources” (i.e., not including those patients referred from BCH or ANC). The other sources include referrals from General Practice (n=33), referrals from GUM clinics (n=15), referrals from other hospital specialty departments (n=13), referrals from Occupational Health (n=9), asylum seekers (n=16), and referral source could not be identified for 18 patients. Pairwise comparisons showed that loss to follow-up was less likely for patients referred from Occupational Health Departments in comparison with those referred from GP practices or GUM clinics.
